# Prevalence, associated factors and clinical features of congenital syphilis among newborns in Mbarara hospital, Uganda

**DOI:** 10.1186/s12884-020-03047-y

**Published:** 2020-07-02

**Authors:** Sam Oloya, David Lyczkowski, Patrick Orikiriza, Max Irama, Yap Boum, Richard Migisha, Julius P. Kiwanuka, Juliet Mwanga-Amumpaire

**Affiliations:** 1 Mbarara University of Science and Technology, P O Box 1410, Mbarara, Uganda; 2 Department of Pediatrics, Newton-Wellesley Hospital, 2014, Washington Street, USA; 3 Epicentre Mbarara Research Centre, P.O. Box 1956, Mbarara, Uganda

**Keywords:** Syphilis; congenital syphilis, Adverse outcome, Stillbirth

## Abstract

**Background:**

While congenital syphilis is a significant public health problem that can cause severe disabilities, little is known about the situation in Uganda. We describe prevalence, associated factors and clinical presentation of congenital syphilis in Mbarara, Uganda.

**Methods:**

A cross sectional study was carried out among mother- newborn dyads from the postnatal ward of Mbarara Regional Referral Hospital (MRRH). After obtaining informed consent, a structured questionnaire was used to capture data on risk factors for congenital syphilis. A finger prick was performed on the mothers for Treponema Pallidum Haemagglutination Assay (TPHA). If TPHA was positive, a venous blood sample was collected from the mother to confirm active infection using Rapid Plasma Reagin (RPR). Venous blood was drawn from a newborn if the mother tested positive by TPHA and RPR. A newborn with RPR titres 4 times higher than the mother was considered to have congenital syphilis. We fit logistic regression models to determine factors associated with congenital syphilis.

**Results:**

Between June and September 2015, we enrolled 2500 mothers and 2502 newborns. Prevalence of syphilis was 3.8% (95% CI 3.1–4.6) among newborn infants and 4.1% (95% CI 3.4–5.0) among their mothers. Maternal age <25 years, past history of genital ulcer, a past history of abnormal vaginal discharge, and not receiving treatment of at least one of genital ulcer, genital itching, lower abdominal pain and abnormal vaginal discharge in the current pregnancy were the risk factors associated with congenital syphilis. The most common clinical feature was hepatosplenomegaly.

**Conclusions:**

We found higher-than-expected syphilis sero-prevalence rates in a high risk population of postnatal mothers and their newborns in Uganda. Bridge populations for syphilis may include mothers not tested during pregnancy, who are usually married and not treated. In accordance with our results, the national policy for syphilis control in Uganda should be strengthened to include universal syphilis screening amongst mother-newborn pairs in postnatal clinics with subsequent partner notification.

## Background

Congenital syphilis is potentially fatal, yet preventable by antenatal screening and treating seropositive pregnant mothers. It manifests itself, according to severity, as late abortion, intrauterine fetal death, stillbirth and low birthweight. Early manifestation of syphilis in the neonatal period include aseptic meningitis, seizures, skin rash and neonatal death. Syphilis may also manifest as latent infection leading to later sequelae. Adverse outcomes are worse in newborns whose mothers have syphilis but have not been treated compared to those born to mothers who received treatment [1, 2]. In 2007, WHO launched the global initiative for the elimination of mother-to-child transmission of syphilis. While the numbers of syphilis-associated pregnancy adverse outcomes decreased from 576.784 cases in 2008 to 350.915 cases in 2012 globally, the reduction was much less in Africa where the number of tested pregnant women decreased, thus increasing risks of transmission to their unborn babies [1, 2]. Untreated, the probability of vertical transmission of syphilis from mother-to-child is 45–70% [3]. Over 90% of the cases with congenital syphilis occurs in low income countries [4], and even though screening for syphilis among pregnant mothers during antenatal care and treating those found positive would be cost effective and avert this situation [5], there are no records of antenatal screening in hospitals in Uganda. Uganda, like many other low resource settings does not have much statistics on the numbers of infected infants or the proportion of pregnant women with syphilis. The few data that exist do not give a full picture how prevalent syphilis is in pregnant mothers, because only 68% of women in low income countries attend antenatal care (ANC) and of these, about half do not attend until after the first trimester. In Uganda, only 47% of pregnant women receive antenatal care and only 42% of births are attended by skilled birth attendants [6, 7]. Even though maternal syphilis screening and treatment are recognized as part of essential antenatal care globally and is included in the Ugandan guidelines for antenatal care, coverage rates are low because of poor availability of screening tests in health facilities and pregnant mothers reporting late in pregnancy for their 1st antenatal visit [8–10]. There is a risk of treatment failure in fetuses of mothers who are diagnosed and have treatment initiated late. In addition treatment in late pregnancy may precipitate the Jarisch-herxheimer reaction resulting into preterm labor and fetal distress [11].

The prevalence of congenital syphilis in Mbarara Regional Referral Hospital (MRRH) is not known; the only available data estimated a 2.2% prevalence of syphilis 20 years ago among women attending ANC [12]. Since congenital syphilis is a sentinel event in antenatal care quality, there is a need for current data. In this study we describe current prevalence and maternal factors associated with congenital syphilis among newborns delivered in MRRH.

## Methods

### Data collection

This was a cross-sectional study of mother-newborn dyads in the maternity ward of MRRH between June and September 2015. They were consecutively enrolled on admission in labour or after birth as long as their newborns were alive. Structured interviewer-administered questionnaires were used to record information including maternal syphilis sero-status in the current and previous pregnancies, history of treatment for syphilis; for those who said they had tested positive, potential risk factors for syphilis infection, including number of sexual partners, marital status and occupation. Significant information related to past obstetric outcomes such as history of late abortions, stillbirths and neonatal deaths were also recorded (Supplement material). When available, the mothers’ antenatal care cards were reviewed to confirm the information.

### Diagnosis of syphilis

Diagnostic testing for maternal Rapid Plasma Reagin (RPR) as confirmatory test of recent infection. We defined active syphilis as blood testing positive with TPHA and RPR. All tests were conducted within 1 h after blood drawing. The approach to diagnostic testing for syphilis in newborn babies followed the CDC 2010 STD Treatment Guidelines [13, 14]. Only newborns of mothers diagnosed positive for syphilis with TPHA and RPR were tested. Newborns’ venous blood was tested using the same RPR test.

RPR titres of both mothers and newborns were measured, and their ratio compared. The newborn’s titre was considered high, if the concentration was four fold higher than that of the mother. Newborns were said to have congenital syphilis if they had high titres of RPR.

### Sample size estimation and analysis

Using sample size estimator in STATA11.0 with Wald approximation, we required to study 2500 mothers, assuming a sero-prevalence rate of 2.5% and transmission rate of 60%, based on a previously reported transmission rate of 30–80% [15]. This would allow detection of a 1.5% prevalence of congenital syphilis with a margin of error of 5%.

Prevalence of congenital syphilis was determined as a proportion of newborns with positive RPRs and clinical features of congenital syphilis or RPR titers fourfold higher than their mothers, relative to all newborns in the study.

Univariate regression analysis was performed to establish common risk factors associated with congenital syphilis. Odds ratios with 95% confidence intervals (CI) were derived for each variable. Variables whose *p*-values were less than 0.4 were included in a multiple logistic regression model to establish those factors that were independently associated with congenital syphilis. Maternal factors included maternal age, history of vaginal discharge during the past or this pregnancy, parity, number of sexual partners during the last year, maternal occupation, genital ulcers prior to or during this pregnancy, previous history of treatment for syphilis and use of antibiotics for any indication during this pregnancy.

## Results

### The mothers

**Fig. 1:**
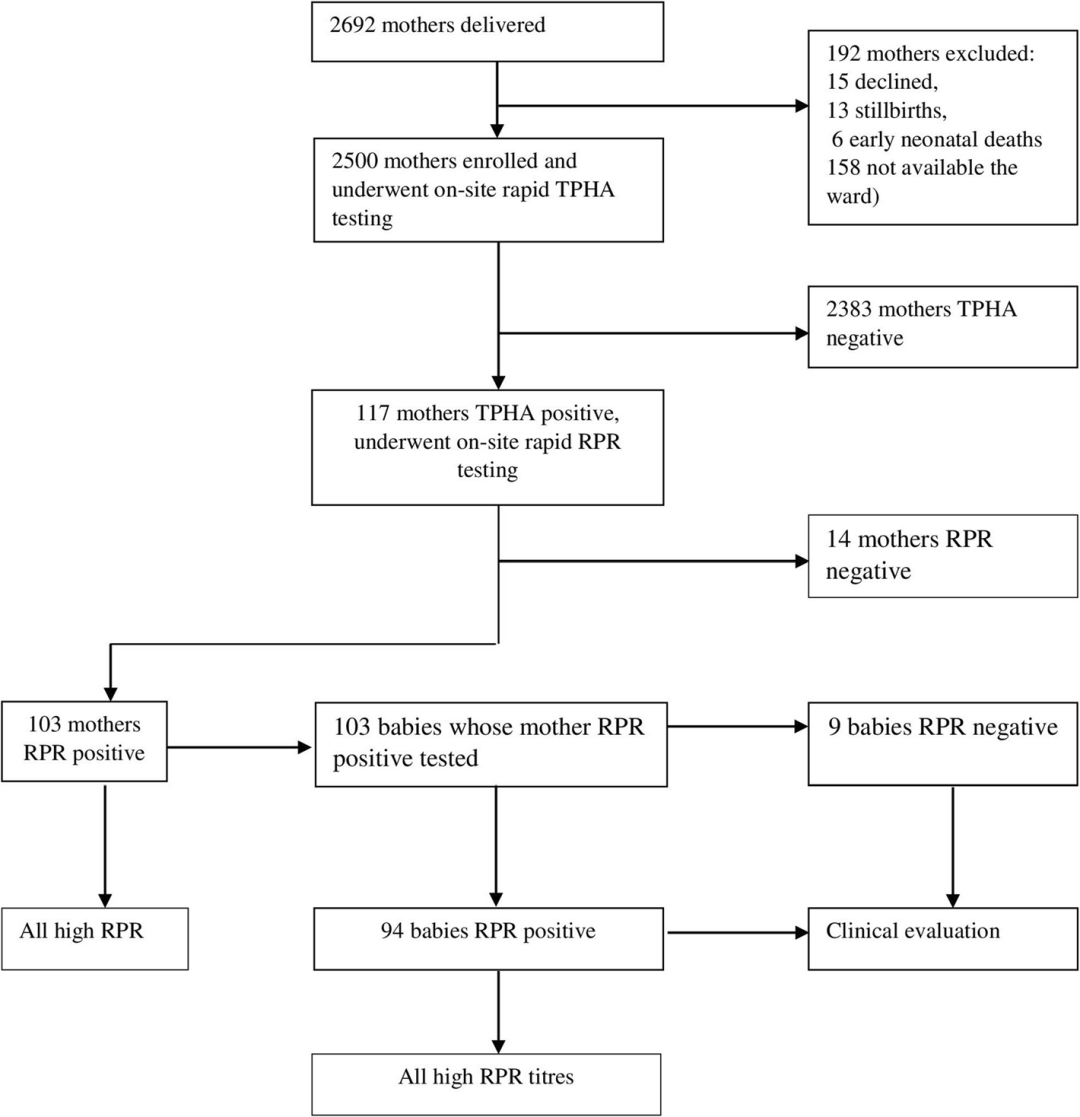
Patients’ flow chart. RPR: Rapid Plasma Reagin. TPHA: Treponema Pallidum Haemagglutination Assay

Between June and September 2015, we enrolled 2500 (median age 20, IQR 15–36 years) out of 2692 eligible mothers; 192 were excluded for declination of consent, stillbirth, perinatal death and discharge before enrollment into the study (Fig. 1). The mothers gave birth to 2502 live babies; including 2 sets of twins. Almost three-quarters of the mothers had been treated for a genital ulcer, vaginal discharge or lower abdominal pain; 1861 (74.5%) in past and 2268 (90.7%) during the current pregnancy (Table 1A in supplement file 1). The majority of mothers were young, multiparous (89.7%) and 88.9% had a single sexual partner (Table 1). Out of the 1159 (54.6%) mothers who reported having tested for syphilis during the current pregnancy, twenty nine (1.2%) had positive RPR results but only 15 (51.7%) reported to have received specific treatment. Twelve (40%) of the spouses of the 29 RPR positive mothers were reported to have received treatment for syphilis. Out of the 117 (4.5%) mothers who tested positive for TPHA, 103 (88.0%) tested RPR positive (Fig. 1).

**Table 1:** Maternal demographic and clinical characteristics and their association with congenital syphilis; univariate analysis

Characteristic	RPR/TPHA (%)		
	Positive (***n*** = 103)	Negative (***n*** = 2397)	OR(95% CI)
**Age in years**			
≤ 24	32 (31.1)	481 (20.1)	**1.73 (1.12–2.69)**
25–35	63 (61.2)	1647 (68.7)	REF
> 35	8 (7.8)	269 (11.2)	0.78 (0.37–1.64)
**Parity**			
1	15 (14.6)	241 (10.1)	1.51 (0.85–2.66)
2–4	76 (73.8)	1840 (76.7)	REF
≥ 5	12 (11.6)	316 (13.1)	0.92 (0.50–1.71)
**No of sexual partners in the last 1 year**			
> 1	3 (2.9)	68 (2.8)	1.07 (0.33–3.46)
declined to respond	3 (2.9)	203 (8.5)	0.36 (0.11–1.14)
1	97 (94.2)	2123 (88.7)	REF
**Occupation**			
Unemployed	38 (36.9)	745 (31.1)	1.26 (0.77–2.05)
Salaried earner	18 (17.5)	544 (22.7)	0.63 (0.29–1.39)
Business	9 (8.7)	320 (13.4)	0.84 (0.46–1.51)
Subsistence	38 (36.9)	788 (32.9)	REF
**History of Genital ulcer**			
No	68 (66.0)	1702 (71.2)	REF
Yes	35 (34.0)	689 (28.8)	1.27 (0.84–1.93)
**History of vaginal discharge**			
No	35 (34.0)	1286 (53.7)	REF
Yes	68 (66.0)	1111 (46.4)	**2.24 (1.45–3.45)**
**History of lower abdominal pain**			
No	45 (43.7)	906 (37.8)	1.28 (0.86–1.90)
Yes	58 (56.3)	1491 (62.2)	REF
**History of treatment for at least one of genital ulcer, genital itch, vaginal discharge or lower abdominal pain in current pregnancy**			
No	27 (26.2)	205 (8.5)	**3.82 (2.41–6.06)**
Yes	76 (73.8)	2192 (91.5)	REF
**Antibiotic use**			
No	79 (76.7)	1797 (75.0)	REF
Yes	15 (14.6)	270 (11.3)	1.26 (0.72–2.23)

*REF* reference category*, OR* odds ratio*, CI* confidence interval

### The infants

During the same period, 2502 newborns were enrolled, median gestational age 39 (IQR 38–40 weeks); 234 (9.4%) were premature and 25 (1%) post term. Ninety four newborns were RPR positive, giving an overall prevalence of congenital syphilis amongst live newborns of 3.8% (95% CI 3.1–4.6). Clinical features of congenital syphilis among 103 babies born to mothers with syphilis are presented in Table 2. The most frequent physical signs were splenomegaly (58.3%) and hepatomegaly (52.4%). More specific cutaneous signs like condylomata lata and petechiae were extremely rare (Tables 2 and 3).

**Table 2:** Clinical features of congenital syphilis among newborns of the 103 mothers with syphilis

Characteristics	Total (n = 103)
	n (%)
Condylomata lata	1 (1.0%)
Syphilitic rash	17 (16.5)
Desquamation	17 (16.5)
Rhinitis	2 (1.9)
Hepatomegaly	54 (52.4)
Splenomegaly	60 (58.3)
Pseudoparalysis	3 (2.9)
Jaundice	3 (2.9)
Anemia	0 (0.0)
Petechiae	2 (1.9)

**Table 3:** Characteristics of RPR Positive and Negative newborns of 103 mothers with syphilis

Characteristic	RPR Negative neonates *n* = 9	RPR Positive neonates *n* = 94
Gestational age in weeks, mean(SD)	39.6 (1.9)	38.8 (1.5)
Birthweight in kg, mean(SD)	3.5 (0.7)	3.3 (0.5)
Temperature, mean(SD)	37.0 (0.4)	37.0 (0.7)
Apgar score, mean(SD)	7.9 (0.8)	6.7 (1.2)
Head Circumference in cm, mean(SD)	36.4 (1.2)	36.3 (0.9)
Splenomegaly, n (%)	0 (0.0)	60 (63.8)
Hepatomegaly, n (%)	2 (22.2)	52 (55.3)
Syphilitic rash, n (%)	0 (0.0)	17 (18.1)

In unadjusted analysis, newborns more likely to acquire congenital syphilis were those born to mothers < 25 years of age, mothers with a previous history of vaginal discharge, and those who had not received any treatment for at least one of genital ulcer, genital itch, vaginal discharge or lower abdominal pain in the current pregnancy (Table 1). In adjusted analysis, maternal age <25 years (aOR 1.85; 95%CI: 1.17–2.92), previous history of genital ulcers (aOR 1.88; 95% CI: 1.19–2.95), previous history of vaginal discharge (aOR 2.72; 95%CI: 1.74–4.26) and not receiving any treatment for genital ulcers or genital itching (aOR 3.91; 95%CI: 2.44–6.25) were associated with increased risk for congenital syphilis (Table 4).

**Table 4:** Factors associated with congenital syphilis; multivariate analysis

Characteristics	aOR	95% CI
Age		
≤ 24	**1.85**	**1.17–2.92**
25–35	REF	REF
> 35	0.72	0.31–1.67
Previous history of genital ulcer, yes	**1.88**	**1.19–2.95**
Previous history of abnormal vaginal discharge, yes	**2.72**	**1.74–4.26**
History of treatment of at least one genital ulcer, genital itching, abdominal pain and vaginal discharge, in current pregnancy, no	**3.91**	**2.44–6.25**

## Discussion

The prevalence of congenital syphilis in Mbarara Regional Referral Hospital of 3.8% is higher than previously reported elsewhere. A report that utilized data from routine testing of pregnant women in 2016, revealed a much lower estimate of 1119 cases per 100,000 live births in general in Africa [16]. The prevalence of 2.7% reported in Belarus [10], 0.03% in USA among blacks and 0.008% among Hispanics, are lower than in MRRH [17]. This is not surprising considering that our population is of a lower socioeconomic status with higher prevalence of maternal syphilis and weak implementation of screening and treatment policies of maternal syphilis during antenatal care (ANC), compounded by the fact that a big percentage of mothers does not attend ANC in early pregnancy [16]. The high prevalence of syphilis (4.1%) in mothers in this study, comparable to 4.0% in Entebbe hospital in Uganda [18], but higher than the 1.0% global prevalence in 2017, resonates with the high prevalence of congenital syphilis in MRRH [19]. High rates of HIV infection in Mbarara (7.9%) could be an underlying reason, because both conditions have common modes of transmission [20].

Maternal age less than 25 years was associated with congenital syphilis as previously described in Ethiopia [21]. In contrast, studies in Nigeria and Tanzania found no significant association with maternal age [22]. Women in a younger age category are in the phase of sexual initiation, which may imply early and unprotected sex with multiple sexual partners, and high numbers of sexually transmitted diseases while older women may have had a chance of previous counseling and treatment [23].

Consistent with other studies, genital ulcers were associated with congenital syphilis. Genital ulcers, a manifestation of early syphilis, bear a high risk of maternal-fetal transmission while treatment of symptomatic mothers was protective [24].

Gaps still exist in our setup to prevent congenital syphilis. Despite WHO and Uganda Ministry of Health guidelines for routine ANC syphilis screening, screening rates remain low [19]. Many facilities offer ANC without guidelines, often without trained staff [9]. Treatment rates among women with syphilis were low. Health system factors such as regular stock outs and inadequate storage conditions for diagnostic kits and medicines and limited technical skills of health workers could explain these gaps, especially in primary health care facilities [8]. These are compounded with poor health seeking behaviors, poverty and traditional beliefs.

The most common clinical features of congenital syphilis, hepatosplenomegaly, are also common signs of other congenital infections [13]. Lack of specificity of these signs underscores the need for routine screening for mothers and if found positive, testing of infants in order to institute treatment timely, knowing that lack of early treatment may result in severe sequelae.

### Limitations

Our study has some limitations. First, confirmatory tests such as dark-field microscopy, immunofluorescence could not be done due to limited resources. Nonetheless, we tested the mothers with a combination of non-treponemal (RPR) and treponemal (TPHA) tests to ensure the reliability of the results. Second, stillbirths were excluded from the study; although it is a recognized complication of syphilis during pregnancy. This could have led to underestimation of prevalence of maternal syphilis.

## Conclusion

Prevalence of congenital syphilis is still high in this region despite global efforts for its eradication. This underscores the need for increased advocacy and implementation of existing guidelines at national level.

## Supplementary information

**Additional file 1.** Prevalence, associated factors, and clinical features of Congenital Syphilis among newborns in Mbarara Regional Referral Hospital, South Western Uganda.

**Additional file 2.** Maternal demographic and clinical characteristics and their association with congenital syphilis; univariate analysis.

## Data Availability

The datasets during and analysed during this study are available from the 1st author on reasonable request.

## Abbreviations

ANC: Antenatal care
AIDS: Acquired immunodeficiency syndrome
CDC: Centre of disease control
HIV: Human immunodeficiency virus
MRRH: Mbarara Regional Referral Hospital
RPR: Rapid Plasma Reagin
STI: Sexually transmitted infection
TPHA: Treponema pallidum haemagglutination assay
UNICEF: United Nations Children’s Fund
VDRL: Venereal disease research laboratory
WHO: World Health Organization
