# Supplementary material for: Prevalence, associated factors and clinical features of congenital syphilis among newborns in Mbarara hospital, Uganda
Source: BMC Pregnancy Childbirth. 2020 Jul 2;20:385. doi: 10.1186/s12884-020-03047-y (PMC7330944; doi:10.1186/s12884-020-03047-y)
Supplement: Supplementary file 1 — Additional file 1. Prevalence, associated factors, and clinical features of Congenital Syphilis among newborns in Mbarara Regional Referral Hospital, South Western Uganda. [file 12884_2020_3047_MOESM1_ESM.pdf]

Appendix i: Data collection tool

PREVALENCE, ASSOCIATED FACTORS AND CLINICAL FEATURES OF CONGENITAL SYPHILIS AMONG NEWBORNS IN MBARARA REGIONAL REFERRAL HOSPITAL, SOUTH WESTERN UGANDA

About the newborn

A Demographic

1-Study number ----------------------------

2-Date of enrolment (dd/mm/yyy)----/----/------

3-Child initial-----------------------------------

4-Date of birth (dd/mm/yyyy)----/------/-----

5-Gender

a) Male ☐

b) Female ☐

6- Birth weight--------------------Grams

7-Gestational age by:

a) Dates--------------weeks

b) Score----------

B-Results of laboratory tests (child)

8-RPR test positive

a)Yes ☐

b) No ☐

9-If positive, RPR titres------------------

C- Child clinical presentation

10-Head circumference ----------------cm

11- Temperature -------------------------Degree C

12- Condylomata

Yes ☐

No ☐

13-Syphilitic skin rash

Yes ☐

No ☐

14-Desquamation

Yes ☐

No ☐

15-Rhinitis

Yes ☐

No ☐

16-Mucosal lesion

Yes ☐

No ☐

17- Lymphadenopathy

Yes ☐

No ☐

18-Hepatomegally

Yes ☐

No ☐

19-Splenomegaly

Yes ☐

No ☐

20-Pseudoparalysis

Yes ☐

No ☐

21-Oedema

Yes ☐

No ☐

22-if yes specify--------------------------------

23-Anaemia

Yes ☐

No ☐

24-Jaundice

Yes ☐

No ☐

25-Petechiae

Yes ☐

No ☐

26-Respiratory distress

Yes ☐

No ☐

27-Dysmorphic feature (facial)

Yes ☐

No ☐

28-If yes then specify-----------------------------

29-Other

Yes ☐

No ☐

30-If yes specify-------------------------

About the mother

A Demographic

1-What is your address?

Village--

Sub-county--

District of residence------------------------------

Nationality--

2- How old are you? State-------------Years

3-What is your marital status?

Cohabiting ☐

Married monogamous ☐

Married polygamous ☐

Widowed ☐

Divorced/separated ☐

4- What is your parity? State----------------

5-Have you ever had the following (tick)

Premature ☐

Still birth ☐

Early neonatal death ☐

5-What is your religion?

Catholic ☐

Protestant ☐

Islamic ☐

Others specify-----------------------------------

6- What is your tribe? ---------------------

7- What is highest level of education attained?

None ☐

Primary ☐

Secondary ☐

High school ☐

Tertiary and above ☐

8-What is your occupation? ☐

Unemployed ☐

Subsistence farming

Salaried employment ☐

Business ☐

Others, specify----------------------------------

B-Past medical history

9-Have you ever tested for syphilis before?

Yes

No

10- if yes what was the result

Positive

Negative

11-If positive, did you get treatment?

Yes

No

11-Have you ever experienced the following (tick)?

Genital ulcer

Vaginal discharge

Lower abdominal pain

Genital itching

12-Did you receive treatment for it?

Yes

No

I don’t remember

C Pregnancy

13-Did you attend ANC?

Yes

No

14-If Yes, from where

Private ☐

HC111 ☐

HC1V ☐

District health hospital ☐

Tertiary Hospital ☐

15-Have you ever experienced the following (tick) during this pregnancy?

Genital ulcer ☐

Vaginal discharge ☐

Lower abdominal pain ☐

Genital itching ☐

16-Did you receive treatment for it?

Yes ☐

No ☐

I don’t remember ☐

17- Did you test for syphilis during this pregnancy?

Yes ☐

No ☐

18- If yes what trimester?

First trimester ☐

Second trimester ☐

Third trimester ☐

19-What was the result?

Positive ☐

Negative

I don’t know

20-Did you receive therapy for syphilis during this pregnancy?

Yes

No

21-if yes, how many days did you get treatment?

State the number of days -----------------------------days

22-Did your spouse also receive the therapy for syphilis?

Yes

No

23-Did you receive any antibiotics during this pregnancy?

Yes

No

I don’t remember

24-if yes, state the antibiotics used-----------------

25-For the last 2 years, how many different sexual pattern have you had sex with?

One only

More than one

I decline

C Laboratory test

25-On-site TPHA test positive?

Yes

No

26-is on-site RPR test positive?

Yes

No

27-state the RPR titre---------------------------
