# Supplementary material for: Prevalence, associated factors and clinical features of congenital syphilis among newborns in Mbarara hospital, Uganda
Source: BMC Pregnancy Childbirth. 2020 Jul 2;20:385. doi: 10.1186/s12884-020-03047-y (PMC7330944; doi:10.1186/s12884-020-03047-y)
Supplement: Supplementary file 2 — Additional file 2. Maternal demographic and clinical characteristics and their association with congenital syphilis; univariate analysis. [file 12884_2020_3047_MOESM2_ESM.docx]

|  | **Overall (N=2,500)** | **RPR/TPHA (%)** | |  |
| --- | --- | --- | --- | --- |
| **Characteristic** | **n/N (%)** | **Positive (n=103)** | **Negative (n=2397)** | **P value** |
| **Age in years** |  |  |  | 0.021 |
| ≤24 | 513(20.5) | 32(31.1) | 481(20.1) |  |
| 25-35 | 1710(68.4) | 63(61.2) | 1647(68.7) |  |
| >35 | 277(11.1) | 8(7.8) | 269(11.2) |  |
| **Parity** |  |  |  | 0.324 |
| 1 | 256(10.2) | 15(14.6) | 241(10.1) |  |
| 2 -4 | 1916(76.6) | 76(73.8) | 1840(76.7) |  |
| ≥5 | 328(13.1) | 12(11.6) | 316(13.1) |  |
| **No of sexual partners in the last 1 year** | | | | 0.104 |
| >1 | 71(2.8) | 3(2.9) | 68(2.8) |  |
| declined to respond | 206(8.3) | 3(2.9) | 203(8.5) |  |
| 1 | 2220(88.9) | 97(94.2) | 2123(88.7) |  |
| **Occupation** |  |  |  | 0.227 |
| Unemployed | 783(31.3) | 38(36.9) | 745(31.1) |  |
| Salaried earner | 562(22.5) | 18(17.5) | 544(22.7) |  |
| Business | 329(13.2) | 9(8.7) | 320(13.4) |  |
| Subsistence | 826(33.0) | 38(36.9) | 788(32.9) |  |
| **Married/Living with partner, yes** | 1785(71.4) | 82(79.6) | 1703(71.1) | 0.464 |
| **Had preterm births, yes** | 40(1.6) | 3(2.9) | 37(1.5) | 0.226 |
| **Had still births, yes** | 115(4.6) | 5(4.9) | 110(4.6) | 0.810 |
| **Had perinatal deaths, yes** | 172(6.9) | 7(6.8) | 165(6.9) | 0.973 |
| **History of Genital ulcer, yes** | 724(29.0) | 35(34.0) | 689(28.8) | 0.258 |
| **History of vaginal discharge, yes** | 1321(52.8) | 68(66.0) | 1111(46.4) | <0.001 |
| **History of lower abdominal pain, yes** | 1549(62.0) | 58(56.3) | 1491(62.2) | 0.228 |
| **Treatment for genital ulcer, vaginal discharge or lower abdominal pain in current pregnancy, yes** | 2,268(90.7) | 76(73.8) | 2192(91.5) | <0.001 |
| **Past history of treatment for genital ulcer, vaginal discharge or lower abdominal pain, yes** | 1861(74.5) | 70(68.0) | 1791(74.8) | 0.289 |
| RPR: *Rapid plasma reagin*; TPHA: *Treponema pallidum* *Hemaglutination Asssay* | | | | |

Table 1A: Detailed table for maternal demographic and clinical characteristics and their association with congenital syphilis; univariate analysis

up
